# RT-qPCR assay for detection of mink astrovirus in outbreaks of diarrhea on Danish mink farms

**DOI:** 10.1371/journal.pone.0252022

**Published:** 2021-05-26

**Authors:** Sofie Barsøe, Karin Ullman, Mikael Leijon, Kjell Olof Hedlund, Jonas Klingström, Louise Iuel Krarup, Lars Andresen, Michelle Lauge Quaade, Anne Sofie Hammer

**Affiliations:** 1 Department of Veterinary and Animal Sciences, Faculty of Health and Medical Sciences, University of Copenhagen, Frederiksberg C, Denmark; 2 Department of Microbiology, National Veterinary Institute, Uppsala, Sweden; 3 Public Health Agency of Sweden, Solna, Sweden; 4 Center for Infectious Medicine, Department of Medicine Huddinge, Karolinska Institutet, Karolinska University Hospital, Stockholm, Sweden; National Institute of Animal Biotechnology, INDIA

## Abstract

Diarrhea in mink kits is a major cause of disease and mortality in the mink production. The etiology remains unknown in most outbreaks due to a lack of diagnostic assays. In the current study we present an RT-qPCR method to detect mink astrovirus in fecal samples from mink kits with diarrhea. All sampled animals were classified based on age and patoanatomical evaluation as having pre-weaning diarrhea, diarrhea in the growth period or as having no macroscopic signs of diarrhea. Fecal samples were analyzed for MiAstV with RT-qPCR, next generation sequencing and electron microscopy in parallel. Mink astrovirus was detected with RT-qPCR in 92 out of 203 samples. This detection was confirmed by next generation sequencing in a high proportion of samples (22/27), and by visualization of astrovirus particles with EM in some of the samples. Mink astrovirus was highly prevalent (68%) among kits in the outbreaks of pre-weaning diarrhea, in particular outbreaks from May, while less prevalent in outbreaks in June. Mink astrovirus was detected in outbreaks of diarrhea in the growth period, though in a much lesser extent than in the pre-weaning period. The role of mink astrovirus in the diarrhea disease complex of mink remain to be investigated, and for that purpose this sensitive and robust RT-qPCR can be a valuable tool in the future.

## Introduction

Diarrhea in mink kits is a major cause of disease and mortality in the mink production [1, 2]. The disease often occurs in outbreaks with a high morbidity and can be associated with stunted growth, reduced pelting quality and/or death of the animals [1, 3, 4]. Thus, diarrhea is a welfare problem as well as an economical problem. Little is known about the involved pathogens, diagnostic possibilities are limited and only few systematic studies of the etiology of diarrhea in mink have been reported over the last decades [3, 5, 6]. Mink Astrovirus (MiAstV) has been proposed as a causative factor in pre-weaning diarrhea and has been epidemiologically linked to outbreaks by detection with electron microscopy (EM) [5] and it has been molecularly characterized [7, 8]. In a larger study of mink with pre-weaning diarrhea astrovirus was detected with next generation sequencing, although other both viral and bacterial agents were also detected [6]. Astrovirus are small (28–30 nm) non-enveloped positive stranded RNA viruses known from other species where it, either as a primary agent or a co-factor, is associated with neonatal diarrhea [9, 10]. Several pathogens in mink are known to have diarrhea either as a primary or secondary clinical sign. Mink enteritis virus, a parvovirus, causes a severe necrotizing enteritis and mortality in the post weaning period [11, 12]. Coronavirus have been detected by EM and PCR in American farm mink with diarrhea in the post weaning period and serological surveys on Danish farms have indicated a high prevalence of mink positive for antibodies specific for coronavirus [13–15]. Other viruses, including calici- and rotavirus, have been suggested to be causal factors of diarrhea in mink kits [6, 11, 13, 15–17]. Additionally, the viral diseases Aleutian disease and distemper can also manifest in the intestinal tract with diarrhea as a clinical sign. These two pathogens were notifiable in Denmark at the time of the study, and has in general been studied more thoroughly, with PCR assays available for both [18, 19]. Naturally, bacteria can also cause diarrhea in mink kits, either as a primary or secondary complication. However, with a focus on detection of virus, it is without the scope of this paper to cover the literature on bacterial etiologies.

In order to prevent and/or treat diarrhea in mink kits it is important to characterize the disease complex fully and develop reliable and available diagnostic tools. In the current study we present and apply a MiAstV specific RT-qPCR to clinical samples to test the performance of the assay and to determine the prevalence of MiAstV in pre- and post-weaning mink kits with diarrhea. Samples from diarrheic animals submitted for necropsy at University of Copenhagen through May until November 2013 were analyzed. The animals were subject to macroscopic necropsies and fecal samples were collected and tested in parallel with RT-qPCR, next generation sequencing (NGS) and EM to confirm the results from the RT-qPCR. We here provide and compare the results of the mentioned analysis and discuss the application of the assay and the findings in relation to the macroscopic pathologic diagnosis.

## Materials and methods

### Animals

A total of 203 mink kits from 44 Danish farms were included in the study (complete overview in S1 Table). Batches of mink kits were received from 39 farms with diarrhea (177 mink kits) and 5 control farms (26 mink kits). Control farms had no history of diarrhea and sampled kits did not have symptoms of diarrhea at the time of submission. The animals were submitted in two periods: the pre-weaning period (May and June, 0–2 months old, total 115 kits (109 case and 6 controls)) and the post-weaning period (July-November, 3–6 months old, 88 kits (68 cases and 20 controls), from here on referred to as the “growth period”). Submission of mink kits from farms with outbreak of diarrhea was voluntary and with no considerations to geographical area, farm size or previous outbreaks.

The mink carcasses included in the study were submitted for post mortem diagnostic investigation at University of Copenhagen. No animals were culled for research purposes, thus no ethical or laboratory animal permission was required. If the animal was euthanized, it was done by trained personal on the farm according to Danish legislation (BEK nr. 135 af 14/02/2014).

### Necropsy and sampling

All animals underwent a full necropsy after delivery to the pathology department at University of Copenhagen. During the necropsy three tubes of feces or a piece of colon (if no more feces were available) were sampled for RT-qPCR, NGS and EM. The samples were frozen (-20°C) immediately after the necropsy until further processing as described below. All submitted animals were from farms with Aleutian disease free status, however the samples for RT-qPCR were also tested for Aleutian disease virus as described by Jensen et al. (2011) [18] and found negative.

The animals were classified in three groups either as having pre-weaning diarrhea (PWD) or growth period diarrhea (GPD) or no macroscopic signs of diarrhea. All animals with PWD had oily to watery diarrhea, excessive exudates on the neck, body, legs and claws, edematous and hyperemic anal region and/or alopecia and were from 0–2 months old. Animals with GPD had either loose or watery stool, fecal contamination of the anal region and intestinal lesions such as edema, hyperemia or hemorrhage and were from 3–6 months old.

### Assay design

Based on sequence data for MiAstV in the GenBank database (accession numbers AY196095-196103 and AY179509) primers and probe were designed targeting the conserved RNA-dependent-RNA-polymerase gene and selected using the software Beacon designer 2.0 (PREMIER Biosoft International, Palo Alto, CA, USA). The sequences of the MiAstV primers and probe were: MiAstV-F_3549: 5´-TCT TRA TGC YCA GTG GTG AGG T-3´, MiAstV-R_3649: 5´-CTG GAA GAA CAC GTT GCA CAA AT-3´, MiAstV_3572 Probe: 5´-ACC CGY CAG ACC AAG GGC AAY CCA-3´. PCR products from 6 positive samples were purified and send to Eurofins Genomics (Eurofins Genomics, Köln, Germany), for standard Sanger sequencing. Obtained sequences confirmed specificity of the assay.

### Extraction of RNA

RNA extraction was performed using Roche High Pure Viral Nucleic Kit, Catalog #11 858 874 001, version 18 according to the manufacturer’s instruction. Briefly, 50 mg sample was mixed with 200 μl TE buffer. If the sample was liquid 50 μl sample and 150 μl TE buffer was used instead. 100 μl 1 mm silica beads were added and the mixture was homogenized. A working solution of 4 μl poly(A) and 200 μl binding buffer pr. sample was mixed, and 200 μl of this solution and 50 μl protein kinase was added to the samples and mixed thoroughly. Incubation in 10 min. at 72°C. After incubation, 100 μl binding buffer was mixed in and the samples were centrifuged at 8000×g for 1 minute. The liquid part of the sample was transferred to a High Pure Filter Tube and centrifuged at 8000×g for 1 minute. The Filter Tubes were transferred to Collection Tubes, 500 μl inhibitor Removal buffer was added and the sample was centrifuged at 8000×g for 1 minute. Hereafter the filter tubes were transferred to new Collection Tubes, 450 μl wash buffer was added and the sample was centrifuged at 8000×g for 1 minute. The Collection Tubes were emptied using a mechanical suction and the centrifugation was repeated. The Filter Tubes were then transferred to Eppendorf tubes, 50 μl elution buffer was added, and the samples were centrifuged at 8000×g for 1 minute. The extracted RNA was marked with sample number and stored at -20°C until further analysis.

### cDNA reaction

cDNA was synthesized by mixing 4 μl of extracted RNA with 4 μl Quanta Biosciences qScript cDNA SuperMix, Cat. No. 95048–100 and 12 μl RNAse free water. The mix was run in a thermal cycler for 5 minutes at 25°C, 30 minutes at 42°C, 5 minutes at 85°C and finally stored at 4°C if qPCR was run the same day or at -20°C for later analysis. Before analysis, the cDNA mixture was diluted 10 times with RNase free water.

### qPCR

**Fig 1:**
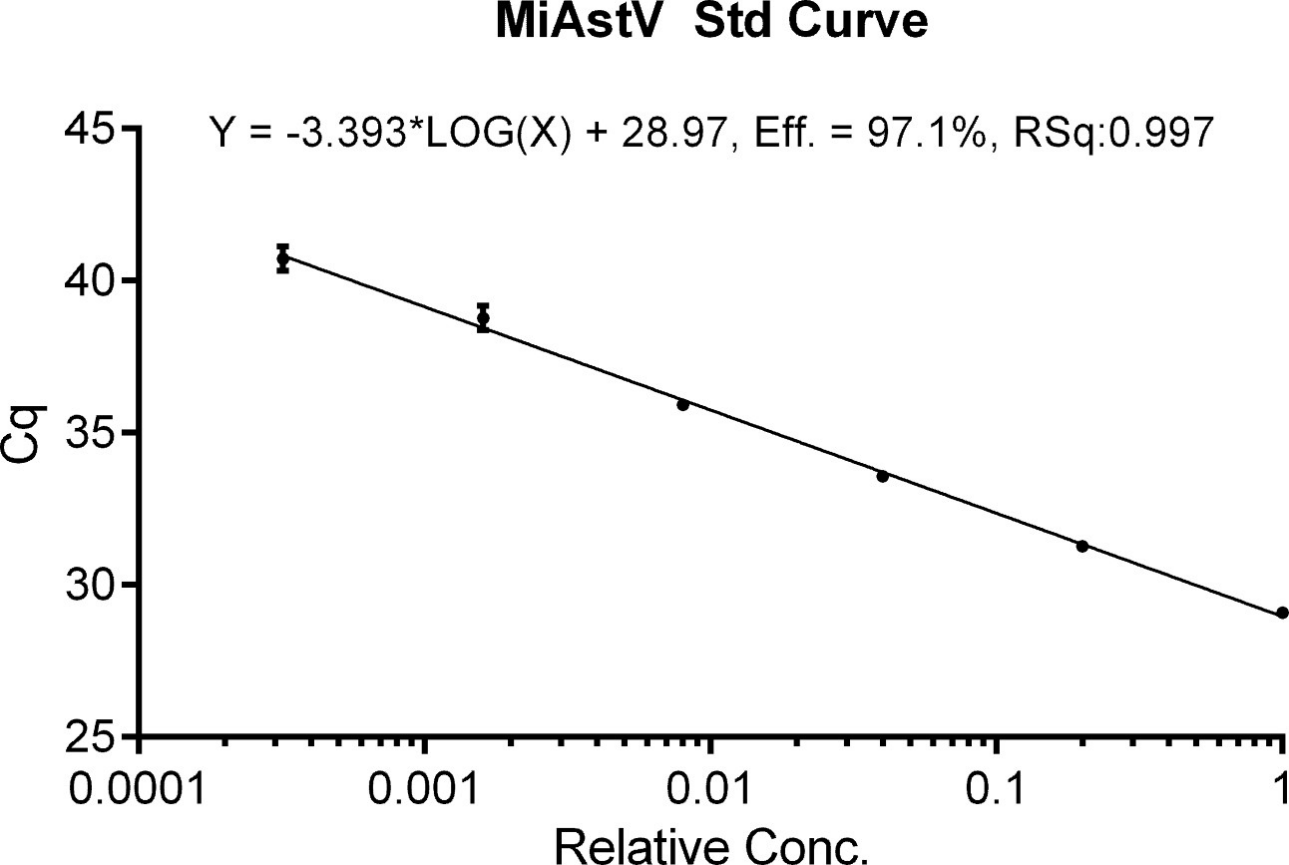
MiAstV std curve. Performance and linearity of the mink astrovirus (MiAstV) qPCR was calculated based on the standard curve of a fivefold serial dilution series using a confirmed positive sample as template.

The qPCR was performed with the commercial kit Quanta Biosciences PerfeCTa qPCR ToughMix low ROX (Catalog #95114–125), on an Agilent Mx3005P thermocycler. The total reaction volume was 20 μl and apart from ToughMix buffer contained 8 pmol of each primer and probe and 5 μl diluted cDNA template. Performance and linearity were assessed using a standard curve (Fig 1), made on a serial dilution of a positive sample (#903, Table 1). Efficiency was calculated by the instrument software MxPro to 97.1% with r2 = 0.998 and with linear range that exceeds at least until Cq = 42. For limit of detection analysis, a synthetic dsDNA Oligo (gBlock) with defined copy number, was purchased from Integrated DNA Technologies (IDT, Leuven, Belgium). The gBlock contained the amplicon defined by the MiAstV primers. Ten-fold dilution series was made and subjected to qPCR. The assay performance showed linearity until 5 copy numbers (data not shown). All samples were run in duplicate and samples with a detectable Cq value in at least one of the duplicate samples were considered positive.

**Table 1:** Positive and negative control samples used in the development of the polymerase chain reaction.

Sample ID	tissue	MiastV
**898**	Feces	Positive
**901**	Feces	Positive
**902**	Feces	Positive
**903**	Feces	Positive
**904**	Feces	Positive
**MiLung**	Lung	Negative

Five MiAstV positive fecal samples and one MiAstV negative lung sample from mink kits (born 2011) were provided by The National Veterinary Institute in Uppsala, Sweden (Table 1). The positive and negative status wes determined using sequencing and electron microscopy as previously described [5].

### Next generation sequencing

The samples for next generation sequencing were shipped on dry ice to the National Veterinary Institute (SVA), Uppsala, Sweden. Feces or a piece of colon from each animal, constituting one pool per farm, was homogenized in 1x DNase buffer (Roche Applied Science) using M tubes in a gentleMACS dissociator (Miltenyi Biotec) with program “RNA_01 for fresh tissue” to prepare a 20% homogenate. After a 3 times freeze-thaw cycle the homogenate was centrifuged at 1000×g for 5 minutes. The supernatant was collected and filtered through a 0.45 μm filter to remove particles of bacterium-size and larger. The filtrate was treated with 400 U/ml of DNase I (Roche Applied Science) and 10 μg/ml of RNase A (Invitrogen) at 37°C for 2 h to degrade unprotected nucleic acids and subsequently RNA was extracted from 200 μl of the nuclease treated samples using a combination of TRIzol (Invitrogen) and RNeasy kit (Qiagen) following the manufactures’ instructions. Sequence independent single primer amplification (SISPA) was applied to extracted RNA as previously described [20]. For a subset of samples (pool 42405, 42406, 42411, 42415, 42424, 42425, 42426, 42479, 42481, 42482, 42511 and 42532) RNA was extracted with EZ1 Virus Mini Kit (QIAGEN) and cDNA was generated without SISPA using SuperScript III first-strand synthesis kit (Invitrogen) according to the manufacturer´s instructions. Double-stranded DNA was obtained by incubation of the cDNA products with Klenow Fragment DNA polymerase (New England Biolabs) at 37°C for 1 h. For both set of samples concentration was measured with a Qubit fluorometer using Qubit dsDNA HS (High Sensitivity) Assay Kit (Invitrogen), and a 0.2 ng/μl aliquot was prepared for each sample. Nextera XT DNA Library Preparation Kit (Illumina, Inc) was used for library preparation according to the manufacturer´s instructions. Agilent High Sensitivity DNA Kit (Agilent) was used to verify the length distribution of the fragments and for quantification of the libraries. Finally, an equimolar amount (2nM) of each sequencing library with sufficient quality and concentration was pooled into four separate library-pools, denatured with NaOH and further diluted with hybridization buffer to a final concentration of 10 pM. Sequencing was performed in four runs on a MiSeq desktop sequencer using MiSeq Reagent Kit v2 500 cycles (Illumina, Inc).

#### Bioinformatics

Illumina MiSeq sequence raw paired-end reads were quality filtered and duplicate reads were removed using HTStream (https://s4hts.github.io/HTStream/). The remaining reads were taxonomically classified with accelerated blastx using DIAMOND version 2.0.6 [21] with a database created from genbank release 241 (Dec 15, 2020). The database was constructed to included taxonomy information and the output file from diamond assigned a ncbi taxid to each read (0 if the read could not be classified). An in-house python script was used to extract the virus reads from the DIAMOND output files. For the set of samples where SISPA was applied, a negative cutoff was set at ≤10 reads.

### Electron microscopy

The samples were analyzed with electron microscopy at the Public Health Agency of Sweden as previously described [5]. A step of high–speed-centrifugation was used for all samples to increase the number of virus particles. Only samples with particles 28 to 30 nm in diameter and with a star-like morphology were considered astrovirus-positive. If the material was deemed too poor, i.e. decomposed, no sample was taken. In total 96 samples from the pre-weaning period and 84 from the growth period period was analyzed.

### Comparison of results

Comparison of the results from RT-qPCR, NGS, pathology and the clinical signs were performed in 2x2 tables and the true agreement, kappa (к), was calculated as $\kappa =\frac{p_{o}{-p}_{e}}{1-p_{e}}$. The kappa value was interpreted using Landis and Koch [22]. The positive predictive value (PPV) and negative predictive value (NPV) was calculated as PPV = number of true positive/(number of true positive+number of false negative) and NPV = number of true negative/(number of true negative + number of false negatives)

## Results

A full overview of the obtained results from the performed analysis are available in S1 Table.

### Pathology

The number of submitted animals and whether these met the criteria set up for PWD and GPD is illustrated in Table 2. All submitted mink were from Aleutian disease free farms, which was confirmed by an Aleutian virus specific qPCR [18] on feces which was negative for all the submitted mink.

**Table 2:** Prevalence of diarrhea in submitted mink.

Submissions	Pre-weaning period^1^	Growth period^2^
Case	96% (105/109)	51% (35/68)
Control	0% (0/6)	30% (6/20)
Total submitted	115	88

^1^Pre-weaning period: 0–2 months old mink kits

^2^growth period: 3–6 months old mink.

### RT-qPCR

MiAstV was detected in 73 out of 109 submissions in the pre-weaning period (May and June). 71 of these positive samples were from kits with PWD and these samples had an average Cq value of 31.2. There is a higher prevalence of positive samples in May (94%) than in June (20%). MiAstV was detected in 17 out of the 68 submissions from the growth period. The average Cq value of these samples were 38.7. In seven cases there were a coincidence of GPD and a positive RT-qPCR result. MiAstV was detected in two (of the 20) control animals, one of these were also GPD positive. Table 3 shows and overview of all positive samples in the two periods, regardless of diagnosis of PWD or GPD.

**Table 3:** Prevalence of mink astrovirus (MiAstV) detected by RT-qPCR.

MiAstV RT-qPCR	^1^Pre-weaning period	^2^Growth period
Case	67% (73/109)	25% (17/68)
Control	0% (0/6)	10% (2/20)
Total animals analyzed	115	88

^1^Pre-weaning period: 0–2 months old mink kits

^2^growth period: 3–6 months old mink.

### Next generation sequencing

NGS was performed on feces pools from 27 farms with at least one RT-qPCR positive mink and 12 farms where all mink were RT-qPCR negative (total 39 farms). MiAstV was confirmed in 22 of the RT-qPCR positive farms, and negativity was confirmed in 6 of the 12 RT-qPCR negative farms (Table 4).

**Table 4:** Results from next generation sequencing grouped in RT-qPCR positive or negative farms.

Journal nr	Mink astrovirus (nr. of non-identical reads)	SISPA^1^ (cutoff ≤10)
**RT-qPCR positive farms (at least one mink with Cq-value)**		
*(p) 42479*	*0*	***-***
*(c) 42481*	*0*	*-*
*42482*	*0*	*-*
*42539*	*2*	*+*
*(p) 42453*	*7*	***+***
*(p) 42425*	*1*	***-***
*(p) 42415*	*2*	***-***
*(p) 42424*	*3*	***-***
*(p) 42411*	*6*	***-***
*42510*	*14*	*+*
*(p) 42438*	*39*	***+***
*(p) 42405*	*125*	***-***
*42508*	*183*	*+*
*(p) 42532*	*204*	***-***
*(p) 42406*	*207*	***-***
*(p) 42465*	*208*	***+***
*42570*	*228*	*+*
*42598*	*263*	*+*
*(p) 42435*	*612*	***+***
*(p) 42436*	*3206*	***+***
*(p) 42413*	*15605*	***+***
*42569*	*20765*	*+*
*42616*	*44254*	*+*
*42596*	*59185*	*+*
*(p) 42416*	*71640*	***+***
*42597*	*108685*	*+*
*(p) 42437*	*112718*	***+***
**RT-qPCR negative farms (no mink with Cq-value)**		
*(c) 42538*	*1*	*+*
*42592*	*3*	*+*
*(p) 42466*	*5*	***+***
*(c) 42480*	*6*	*+*
*42738*	*7*	*+*
*(p) 42467*	*10*	***+***
*(p)(c) 42426*	*4*	***-***
*(p) 42464*	*11*	***+***
*(p) 42454*	*19*	***+***
*42734*	*27*	*+*
*42511*	*74*	*-*
*(c) 42611*	*3453*	*+*

(c) = control farm. (p) = submissions from the pre-weaning period. Red = negative sequencing, green = positive sequencing.

^1^Sequence independent single primer amplification (SISPA) was applied to extracted RNA (marked with “+”) in some of the pools increasing the sensitivity why a negative cutoff at ≤ 10 was applied.

### Electron microscopy

166 samples were successfully analyzed with EM, and both astrovirus and calicivirus was detected. The astrovirus particles usually appeared in aggregates and there were always some particles with the recognizable star-like morphology. Samples with single particles usually less than 28 nm in diameter and without stars were not considered positive for astrovirus. MiAstV was detected with RT-qPCR in 11 out of the 19 mink where astrovirus was detected with EM (Table 5).

**Table 5:** Detection of astro- and calicivirus with electron microscopy in mink feces.

	Analyzed in EM	Astrovirus	Calicivirus
Pre-weaning^1^	86	10 (9)	41
Growth period^2^	80	9 (2)	14
Total	166	19 (11)	55

Number of samples where astro- or calicivirus was detected with electron microscopy. The number in brackets indicate the number of mink that was also positive for mink astrovirus in MiAstV RT-qPCR.

^1^Pre-weaning: 0–2 months old mink kits

^2^growth period: 3–6 months old mink.

### Comparison of anamnesis, pathology, RT-qPCR and NGS

In the following tables (Tables 6–10) a calculation of the agreement between the different methods used (anamnesis, patoanatomical classification, RT-qPCR and NGS) is made as well as the negative predictive value (NPV) and positive predictive value (PPV).

**Table 6:** Comparison of anamnesis and pathological classification of pre-weaning diarrhea.

		+	PWD		-	total	PPV, NPV and к [95% CI]
**Anamnesis**	+	105		4		109	PPV = 0.96 [0.90–0.99]
	-	0		6		6	NPV = 1.00 [0.52–1.00]
**Total**		105		10		115	к = 0.73 [0.48–0.99] SE = 0.13

PWD, pre-weaning diarrhea.К, PPV and NPV is calculated as described in section “Comparison of results”.

**Table 7:** Comparison of anamnesis and pathological classification of diarrhea in the growth period.

		+	GPD		-	total	PPV, NPV and к [95% CI]
**Anamnesis**	+	35		33		68	PPV = 0.51 [0.39–0.64]
	-	6		14		20	NPV = 0.70 [0.46–0.87]
**Total**		41		47		88	к = 0.15 [0–0.35] SE = 0.10

GPD, growth period diarrhea.К, PPV and NPV is calculated as described in section “Comparison of results”.

**Table 8:** Comparison of RT-qPCR result for mink- astrovirus and pathological classification of pre-weaning diarrhea.

		+	PWD		-	total	PPV, NPV and к [95% CI]
**MiAstV RT-qPCR**	+	71		2		73	PPV = 0.97 [0.90–1.00]
	-	34		8		37	NPV = 0.19 [0.09–0.35]
**Total**		105		10		115	к = 0.20 [0.0–0.41] SE = 0.11

PWD, pre weaning diarrhea. К, PPV and NPV is calculated as described in section “Comparison of results”.

**Table 9:** Comparison of RT-qPCR result for mink astrovirus and pathological classification of diarrhea in the growth period.

		+	GPD		-	total	PPV, NPV and к [95% CI]
**MiAstV RT-qPCR**	+	8		9		17	PPV = 0.47 [0.24–0.71]
	-	33		38		71	NPV = 0.53 [0.41–0.65]
**Total**		41		47		88	к = 0.00 [0.00–0.22] SE = 0.11

GPD, growths period diarrhea.К, PPV and NPV is calculated as described in section “Comparison of results”.

**Table 10:** Comparison of detection of mink astrovirus (MiAstV) with RT-qPCR and next generation sequencing.

		+	MiAstV RTqPCR		-	total
**MiAstV NGS**	+	22		6		28
	-	5		6		11
**Total**		27		12		39
**к = 0.32 [0.0–0.66], SE = 0.17**						

NGS, next generation sequencing. К is calculated as described in section “Comparison of results”.

## Discussion

In the pre-weaning period, submitted mink kits had characteristic clinical signs of pre-weaning diarrhea such as excessive exudation and yellow oily or watery diarrhea. This is referred to as “sticky” or “greasy” kits by the farmers and practicing veterinarians because of the clinical signs. This clinical diagnosis/anamnesis was confirmed in almost all kits by pathologic examination (96%, Table 5) and the kappa value of the clinical and pathologic diagnosis showed substantial agreement (к = 0.73 [0.48–0.99], Table 5).

MiAstV was detected with RT-qPCR in 68% (71/105) of the cases of pre-weaning diarrhea. MiAstV was also detected in two (out of four) mink kits that did not show signs of PWD, although these mink kits came from a batch of kits with PWD and MiAstV was detected in the other kits. MiAstV has previously been detected in outbreaks of pre-weaning diarrhea and epidemiologically linked to such outbreaks [5, 8]. The comparison of pathologic signs of pre-weaning diarrhea and presence of MiAstV detected by RT-qPCR showed a slight agreement (0.20 [0.0–0.41], Table 8). This is not as convincing as expected if MiAstV is the cause of the pathologic lesions. The PPV is very high though, indicating, that if MiAstV was detected, pre-weaning diarrhea was diagnosed in the majority of the cases (PPV = 0.97, Table 8). The cases where a patoanatomical diagnosis of PWD are made and no astrovirus is detected are mainly from June, where 30 out of 36 kits with pre-weaning diarrhea were negative for MiAstV. This could indicate that the later submissions have been send in too late in the clinical disease, so that virus has been cleared. However, lesions were present in the same degree and at the same state as earlier submissions, so maybe the reason is that (an)other pathogen(s) are responsible for the outbreaks in the later cases of pre-weaning diarrhea. This is supported by the EM analysis, where calicivirus is found in a large number of the samples (Table 5). Birch et al. (2018) find a high prevalence of calicivirus in mink kits with diarrhea in addition to astrovirus, when they characterized the microbiome of feces from mink kits with and without diarrhea. In addition, they find a shift in the relative composition of intestinal bacteria indicating that also bacteria are involved in or influenced by the disease complex. In an older study, calicivirus has likewise been detected in feces pools from mink kits with diarrhea using EM and also RT-PCR [17], although the authors did not compare with clinical feces samples from healthy mink or eliminate other pathogens (except coronavirus) to be sure the diarrhea did not have another etiology. None the less, these findings support the hypothesis that the pre-weaning diarrhea complex is not a disease in itself but a complex of clinical signs, like diarrhea, that can have different or complex etiologies.

In the growth period, there was only a slight agreement between the anamnesis and the pathological diagnosis of GPD (0.15 [0–0.35], Table 7), indicating discrepancies between what was seen on the farm and what could be seen on the necropsy. The PPV = 0.51 (Table 7) shows that only half of the submitted cases could be confirmed pato-anatomically. If the submitted mink is not representative of the situation on the farm, or if the farm actually has another disease problem with thin feces as a secondary complication, this complicates the diagnostic search for pathogens involved in diarrhea.

In samples from the growth period MiAstV was detected with RT-qPCR (Table 9), although not as prevalent and with a higher Cq value than in the pre-weaning period. Further studies would be needed to investigate the role of MiAstV in the growth period. In other species, such as humans, astrovirus is primarily a pathogen of neonatal enteric disease [9]. The question of the diarrheic status of the mink submitted in the growth period should also be kept in mind, and support the hypothesis that MiAstV is an occasional finding in this period.

NGS confirmed the presence of MiAstV in 22 out of 27 RT-qPCR positive farms, confirming the specificity of the assay. The agreement between the methods was only slight (к = 0.32, Table 10). NGS was done on pooled samples and in general is less sensitive than the very sensitive RT-qPCR, which could explain why the last five RT-qPCR positive farms was not confirmed. On the contrary sequences matching MiAstV was detected in 6 out of 12 RT-qPCR negative farms, questioning the sensitivity of the assay. These were, however, mostly with very low reads (4, 11, 19, 27 and 74 reads) with only one farm having a high read (3453 reads) (Table 4). These were mostly from farms with only one or two RT-qPCR positive mink with Cq > 38 in the late pre-weaning period and the growth period.

Astrovirus was only visualized by EM in very few of the mink where MiAstV was detected by RT-qPCR (Table 5). This is probably attributable to the basic differences in the methods. RT-qPCR is very sensitive, and can detect small amounts of virus RNA, even in poor quality samples where the virions are degraded. EM is dependent on a positive visual detection of astrovirus particles, hence the virions needs to be intact and present in a relatively large amount to be detected [23–25]. In eight cases, primarily in the growth period, astrovirus was detected in EM though not in RT-qPCR (Table 5). For two of the cases, belonging to two different farms, MiAstV was detected with NGS in pools of feces from the farm. In one of the cases, MiAstV was detected with RT-qPCR in another mink from the same farm, although with high Cq value (Cq = 38.26). The three analysis, RT-qPCR, EM and NGS, was performed on three different samples. The first two being similar samples from the same animals and the latter was a pool of all animals from the farm (usually 4–5 animals). The analysis are therefore not performed on the exact same material, and it is possible that the presence of virus vary between the samples. In the growth period the mink eat solid feed composed from fish, meat and slaughter offal, why it is also possible that astrovirus from other sources appear which cannot be morphological distinguished from MiAstV in EM.

We had samples positive for MiAstV in RT-qPCR with Cq values ranging from below 20 to just above 40. This was within the linear range of the RT-qPCR, and the samples were therefore considered positive. However, with the very high Cq values seen in the growth period >38 and the increasing discrepancies between the different analysis suggest it would be relevant with a diagnostic cut-off of the Cq value, to discriminate between samples with a clinical relevant amount of MiAstV and samples with incidental findings of MiAstV. Future studies should be aimed at investigating the diagnostic value of the Cq-value and try to define a relevant cut off.

## Conclusion

Here, we present a robust and sensitive RT-qPCR method for detection of MiAstV and apply it to clinical samples. The detection of MiAst in samples was confirmed by NGS and EM in a large number of the samples, and only in very few cases was MiAstV detected with NGS when not detected with the RT-qPCR assay. We detect a high prevalence of MiAstV in mink kits with preweaning diarrhea, consistent with previous studies [5, 7, 8]. This highlights the possibility of astrovirus as a (co-)factor of diarrhea in pre-weaning mink kits. However, the detection of calicivirus in EM highlight the fact that other viruses may be contributable as well, and this should be investigated further. To our knowledge, this is the first RT-qPCR to detect MiAstV and it will be a valuable tool in future diagnostic investigations of diarrhea in mink kits.

## Supporting information

S1 Table Overview of submitting farms, pathologic investigations, results of RT-qPCR, next generation sequencing (NGS) and electron microscopy (EM). (XLSX)

S2 Table Electron microscopy raw data. (XLSX)
